# Positive Correlation of PTH-Related Peptide with Glucose in Type 2 Diabetes

**DOI:** 10.1155/2009/291027

**Published:** 2009-08-31

**Authors:** Ioannis Legakis, Timos Mantouridis

**Affiliations:** ^1^Department of Endocrinology, Henry Dynant Hospital, 11526 Athens, Greece; ^2^Laboratory of Medical Biopathology, Evgenidion Hospital, Medical School, University of Athens, 11528 Athens, Greece

## Abstract

Type 2 diabetes is characterized by hyperglycemia resulting from insulin resistance in the setting of inadequate beta-cell compensation. Recent studies indicate that for attaining a well-functioning ß-cell mass, parathyroid hormone-related protein (PTHrP) is a very promising candidate among several insulinotropic peptides. In order to elucidate its role, we determine the levels of PTHrP, insulin and c-peptide in type 2 diabetics and in normal subjects in the fasting state. We enrolled 28 patients (16 men and 12 postmenopausal women) with type 2 diabetes and twenty eight aged-matched healthy individuals as control subjects (15 men and 13 women). PTHrP was statistically significant correlated with glucose in type 2 diabetes and in normal subjects in the fasting state. Additionally, PTHrP serum levels exhibited a significant increase in type 2 diabetes compared to control subjects. Interestingly, PTHrP showed a positive correlation with insulin levels only among healthy individuals presumably due to defective glucose stimulated insulin secretion known to occur in type 2 diabetics. In conclusion, the strong positive relation of PTHrP with glucose in the fasting state in patients with type 2 diabetes mellitus raises several questions for further experimentation concerning its exact role and physiological significance.

## 1. Introduction

Blood glucose homeostasis is controlled by the endocrine cells of the pancreas, located in the islets of Langerhans. The islet cells monitor the concentration of glucose in the blood and secrete hormones with opposite effects. Failure of ß-cell survival is critical to the etiology of diabetes mellitus as well as in the setting of islet transplantation [1, 2].

Recently a large number of factors controlling the differentiation of beta-cells have been identified. They are classified into the following main categories: growth factors, cytokine and inflammatory factors, and hormones, such as PTH-related peptide (PTHrP) and glucagon-like peptide-1 (GLP-1) [3–5]. Indeed, mice with PTHrP overexpression under the control of the rat insulin gene promoter derive their increases in ß-cell number and overall islet mass, not as a result of an increase in b-cell proliferation but from a prolongation of ß-cell survival [5]. In general, treatment with these external stimuli can restore a functional beta-cell mass in diabetic animals, but further studies are required before it can be applied to humans.

In that respect, the recognition that PTHrP overexpression enhances ß-cell survival shows potential therapeutic targets for pharmaceutical agents aimed at improving the survival of ß-cells in diabetes [6]. As an attempt to investigate the role of PTHrP in diabetes, we performed serum determinations of PTHrP, insulin, and c-peptide in type 2 diabetics and in normal subjects in the fasting state.

## 2. Materials and Methods

We enrolled 28 patients with type 2 diabetes (HbA1c 7.18 ± : 0.5%) 16 men (aged 56.8 ± 2.4 years, BMI 29 ± 1.9 kg/m2) and 12 postmenopausal women (follicle-stimulating hormone (FSH) >30 mIU/mL, aged 58 ± 2 years, BMI 29 ± 1.7 kg/m^2^) with a maximum disease duration of 4 years. Twenty-eight healthy individuals participated in the study as control subjects: 15 men (aged 57.3 ± 1.3 years, BMI 27.7 ± 1.09 kg/m^2^) and 13 women (FSH >30 mIU/mL, aged 56.84 ± 1.8 years, BMI 27.46 ± 1.3 kg/m^2^) with no history of diabetes, hypertension, liver, or kidney disease. None of the nondiabetic healthy volunteers were taking any medication, and none had a first degree relative with type 2 diabetes.

Written informed consent was obtained from all study participants. Blood samples were collected at rest at 8:00 A.M., after an overnight fast and 24-hour alcohol abstention. PTHrP was determined in serum by a competitive enzyme immunoassay (Peninsula Laboratories, Belmont, CA). Insulin was measured in serum by an enzyme-linked immunosorbent assay (A×SYM; Abbott Laboratories, North Chicago, IL). A two-site sandwich immunoassay, using direct chemiluminescent technology (ADVIA Centaur; Bayer, Leverkusen, Germany), was used for the determination of serum C-peptide. Statistical evaluation of the results was performed using multivariate median regression models. Statistical significance was set at *P* < .05. Confidence intervals (CI) are reported at 95%.

## 3. Results

According to our data, a statistical significant increase was detected in both PTHrP and glucose levels in women and men with type 2 diabetes compared with control subjects. In particular, PTHrP serum levels showed a significant (*P* < .001) correlation between sex and health status. The estimated difference for health status (diabetics versus healthy) on median PTH-related protein levels was 130 pg/mL (95% CI: 92–168, *P* < .001) among male individuals and 230 pg/mL (95% CI: 196–264, *P* < .001) among female individuals (after adjusting for differences of health status).

The estimated difference for sex (male versus female) on median PTH-related protein levels was 10 pg/mL (95% CI: −24–44, *P* = .562) among healthy individuals (non-significant) and 110 pg/mL (95% CI: 72–148, *P* < .001) among diabetics (after adjusting for differences by health status, Table 1). 

Performing Spearman correlation coefficient analysis, positive and statistically significant correlations were detected between (a) glucose and PTHrP and (b) c-peptide and insulin. These positive associations were slightly more pronounced among diabetics. There was no difference in the strength of these associations between men and women (Table 2). Interestingly, PTHrP showed a positive correlation with insulin levels only among healthy individuals presumably due to defective glucose stimulated insulin secretion known to occur in type 2 diabetics (Table 3).

## 4. Discussion

Type 2 diabetes is characterized by hyperglycemia resulting from insulin resistance in the setting of inadequate ß-cell compensation. Impaired ß-cell function and possibly beta-cell mass appear to be reversible, particularly at early stages of the disease. Despite an enormous increase in our understanding of islet differentiation and development, there is sparse information regarding the factors and pathways that regulate growth, survival, and death of islet cells. Nevertheless, in recent years, a large number of factors controlling the differentiation of ß-cells have been identified, and among them the PTHrP emerged as a strong candidate in ß-cell survival.

Ishida et al. [7] reported that type 2 diabetics exhibited higher serum PTHrP levels than control subjects. However, in that study no stimulation tests were done to demonstrate whether PTHrP is released from the pancreas in response to insulin secretagogues like glucose or calcium. Extensive studies have demonstrated that an increase in the cytosolic calcium is essential for glucose-stimulated insulin release [8]. Indeed, one of the mature, secretory forms of PTHrP, was demonstrated by Wu et al. [9] to increase cytosolic calcium levels in a pancreatic *b*-cell line (RIN 1046-38). Increased cytosolic calcium levels have also been reported in insulinoma cells supporting the notion that PTHrP is not only secreted by pancreatic cells, but might also play an autocrine or paracrine role within the islets themselves [10]. In 2006, Shor et al. [11] evaluated the effects of both oral calcium and glucose loads on insulin and PTHrP secretion in healthy controls. They reported that PTHrP and insulin rose are in parallel although this response was not observed during the calcium load. Moreover, they found significant differences in basal serum PTHrP levels particularly in type 2 versus type 1 diabetics and healthy controls. Ishida et al. [7] proposed that elevated PTHrP levels might play a compensatory role in calcium homeostasis in diabetic patients. They speculate that these patients often exhibit osteopenia and lower than normal PTH levels with a net result: the preservation of normal serum calcium levels. Additional evidence was further obtained by the group of Suzuki et al. [12] who found a significant positive correlation between calcemia and PTHrP in noninsulin-dependent diabetic patients.

In line with the previous reports, we confirmed that PTHrP serum levels exhibited a significant increase in type 2 diabetes compared to control subjects. Moreover, our data demonstrate that that PTHrP was statistically significantly correlated with glucose in type 2 diabetes and in normal subjects in the fasting state. Interestingly, PTHrP showed a positive correlation with insulin levels only among healthy individuals presumably due to defective glucose stimulated insulin secretion known to occur in type 2 diabetics.

In conclusion, a strong positive correlation of PTHrP has been established with glucose in the fasting state. PTHrP appears to be related to the presence of type 2 diabetes and not to the patient's obesity and hormonal status. Further studies are needed in order to elucidate the exact role and the physiological significance of PTHrP in patients with type 2 diabetes mellitus.

## Figures and Tables

**Table 1:** Summary of glucose, PTH-related protein, C-peptide, and insulin levels in males and females by health status (healthy or diabetics).

	Female	Male
	Median (IQR)*	Median (IQR)
*Diabetes*		
* *Glucose (mg/dL)	146.5 (138.0, 198.0)	169.0 (150.5, 196.0)
* *PTH-related protein (pg/mL)	300.0 (265.0, 555.0)	430.0 (320.0, 550.0)
* *C-peptide (ng/mL)	2.28 (1.87, 3.38)	2.44 (1.84, 3.62)
* *Insulin (*μ*IU/L)	7.35 (4.80, 11.95)	10.71 (5.75, 17.08)
*Normal*		
* *Glucose (mg/dL)	89.0 (85.0, 97.0)	98.0 (85.0, 106.0)
* *PTH-related protein (pg/mL)	180.0 (170.0, 190.0)	190.0 (180.0, 200.0)
* *C-peptide (ng/mL)	2.22 (2.00, 3.70)	2.13 (1.86, 2.78)
* *Insulin (*μ*IU/L)	7.13 (4.88, 9.13)	8.20 (6.39, 10.99)

*Interquartile range.

**Table 2:** Correlation coefficients (Spearman's Rho, *P*-values in italics) between glucose, PTH-related protein, C-peptide, and insulin (by sex) in diabetics.

	Glucose	PTH-related protein	C-peptide
	R	R	R
	*P*	*P*	*P*
*Female*			
* *PTH-related protein	**0.9732**		
	<**.0001**		
* *C-peptide	0.1781	0.1528	
	.3942	.4658	
* *Insulin	0.1697	0.1594	**0.8671**
	.4173	.4465	<**.0001**
*Male*			
* *PTH-related protein	**0.9905**		
	<**.0001**		
* *C-peptide	0.2078	0.1787	
	.2621	.3361	
* *Insulin	0.3035	0.2949	**0.8246**
	.0970	.1073	<**.0001**

**Table 3:** Correlation coefficients (Spearman's Rho, *P*-values in italics) between Glucose, PTH-related protein, C-peptide, and insulin (by health status).

	Glucose	PTH-related protein	C-peptide
	R	R	R
	*P*	*P*	*P*
*Diabetics*			
* *PTH-related protein	**0.9922**		
	<**.0001**		
* *C-peptide	0.1176	0.1083	
	.5512	.5833	
* *Insulin	0.0385	0.0371	**0.8692**
	.8459	.8511	<**.0001**
*Healthy*			
* *PTH-related protein	**0.9080**		
	<**.0001**		
* *C-peptide	0.3454	0.3095	
	.0718	.1090	
* *Insulin	**0.5095**	**0.4937**	**0.7378**
	**.0056**	**.0076**	<**.0001**
